# Increase in acute mastoiditis at the end of the COVID-19 pandemic

**DOI:** 10.1007/s00405-024-08704-y

**Published:** 2024-05-14

**Authors:** Eva Goldberg-Bockhorn, Clara Hurzlmeier, Julius M. Vahl, Franziska Stupp, Aleš Janda, Heike von Baum, Thomas K. Hoffmann

**Affiliations:** 1 Department of Otorhinolaryngology, Head and Neck Surgery, Ulm University Medical Center, Frauensteige 12, 89075 Ulm, Germany; 2 Department of Pediatrics and Adolescent Medicine, Ulm University Medical Center, Eythstraße 24, 89075 Ulm, Germany; 3 Institute of Medical Microbiology and Hospital Hygiene, Ulm University Medical Center, Albert-Einstein-Allee 23, 89081 Ulm, Germany

**Keywords:** Acute mastoiditis, Acute otitis media, Pneumococcal infection, GAS, Complications, COVID-19

## Abstract

**Purpose:**

Common respiratory infections were significantly reduced during the COVID-19 pandemic due to general protective and hygiene measures. The gradual withdrawal of these non-pharmaceutical interventions (NPI) was associated with a notable increase in these infections, particularly in pediatric and adult otorhinolaryngology. The aim of this retrospective monocentric study was to evaluate the impact of NPI during the COVID-19 pandemic on the incidence and severity of acute mastoiditis (AM).

**Methods:**

Pre-pandemic clinical data of AM cases from 2011 to 2019 were compared with infection counts from January 2020 to June 2023 for seasonal periodicity, age-specific differences, pathogens, and complication rates in a German third-level hospital.

**Results:**

Out of 196 patients with AM 133 were children, the majority between 1 and 5 years of age. Complications of AM, such as meningitis, brain abscess, and sinus vein thrombosis, were more common in adults (87%) than in children (17%). Morbidity and mortality rates were similar before, during and after the pandemic. Pneumococci were the most common pathogen in both age groups, with a post-pandemic cumulation of *Streptococcus pyogenes* infections in children. While pre-pandemic cases clustered in spring, seasonality was absent in all age groups during the main phase of the pandemic. The cessation of NPI caused a steep rise in AM cases in both age groups starting from December 2022.

**Conclusion:**

NPI during the COVID-19 pandemic reduced the incidence of AM. Their reversal led to a substantial increase in the incidence of AM during the post-pandemic period, which may be due to a general increase in viral respiratory infections and an insufficiently trained immune system.

## Introduction

An acute mastoiditis (AM) typically develops from an acute otitis media (AOM) in consequence of an infection of the upper airway [1]. The involvement of the mastoid mucosa in AOM due to the close proximity between those two sites of infection, which are connected via the aditus ad antrum, is common and makes the definition and differentiational diagnosis of AM difficult [2]. Besides individual factors like age, immune status and bacterial flora of the upper respiratory tract, anatomical conditions seem to be particularly important for the development of AM from AOM. The blocking of the antrum by mucosal swelling and granulation tissue hinders the purulent secretion from draining off the mastoid air cells. Consequently, the inflammation spreads by alternative routes and arrosion of the temporal bone occurs, resulting in serious extratemporal and intracranial complications [2]. The most important risk factor for the development of AM is young age [2]. High fever and elevated inflammation levels are strong indicators of complicated otitis media and the development towards AM [2, 3]. In addition, previous antibiotic therapy and previous ear infections might be considered as additional, minor risk factors for AOM to AM transition [2, 3].

AM is mostly caused by *Streptococcus pneumoniae* (*S*. *pneumoniae*), followed by *Streptococcus pyogenes* (*S. pyogenes*) und *Staphylococcus aureus* (*S. aureus*) [2]. Recurrent or chronic otitis and an inserted ventilation tube are common risk factors for the less common infection by *Pseudomonas aeruginosa* (*P. aeruginosa*). However, contamination of the diagnostic swab with pseudomonades from the outer ear canal or co-infection with other, probably more relevant, but undetected pathogens must be considered in the presence of this germ [2, 4, 5].

The possible development of life-threatening complications of AM requires prompt therapeutic intervention [1, 2, 6]. In addition to antibiotic therapy different surgical procedures are needed if the patient fails to recover. Historically, mastoidectomy has been the standard surgical treatment and, according to the literature, the most effective treatment option [1, 6, 7]. The combination with an adenectomy and the insertion of a ventilation tube is common in children. In case of minor symptoms paracentesis and insertion of a ventilation tube alone, accompanied by drug therapy, may be sufficient [1, 2, 8].

As a result of non-pharmaceutical interventions (NPI), such as contact restrictions including lockdown and school closures, more accurate hand hygiene and wearing face masks during the COVID-19 pandemic, a sharp decrease of infections transmitted via the respiratory tract has been reported [9, 10]. Consequently, a worldwide decrease in ENT infections occurred, including AM and otogenic meningitis [11–16]. Following the lifting of NPI, the number of patients with respiratory diseases treated in emergency departments is noticeably rising again [17, 18]. This increase appears to be also reflected in the amount of AM cases treated, although definite data from systematic surveys are lacking to date. Based on monocentric data of all AM cases hospitalized in the ENT department of a German third level hospital from 2011 to June 2023, we will identify and discuss the influence of seasonal and peri-pandemic factors on the incidence rate of AM.

## Methods

A systematic retrospective data analysis in the period from January 2011 to June 2023 was conducted using the medical documentation in the electronic patient files of the Department of Otorhinolaryngology at a third level hospital in Germany. Files with admission diagnosis of “acute mastoiditis” and “mastoiditis, unspecified” were collected and screened for those patients which were hospitalized and showed typical clinical signs of AM or severe complications. These signs of local AOM progression and transition to AM could be redness and swelling behind the ear, protruding auricle, protrusion of the posterior auditory canal wall, and cervical swelling extending from the mastoid tip, always accompanied by AOM with or without otorrhea. Patients without these typical local symptoms primarily presented with severe complications like meningitis. In these cases, underlying AM was confirmed by CT or MRI and the presence of AOM was verified by further clinical examination. In addition to patient-related data (age, sex), information on detected pathogens and inflammatory complications such as otogen meningitis, sinus vein thrombosis, brain abscess, facial nerve palsy, and Bezold’s abscess was collected. Subperiostal abscess (SPA) was not considered a separate complication from AM. The presence of a preexisting chronic otitis media (perforation of tympanic membrane or cholesteatoma) was not an exclusion criterion if directly preceding otorrhea and antibiotic treatment were absent. The study was positively evaluated by the local ethics committee (ethics vote 517/20).

Data were analyzed descriptively. Absolute and relative frequencies were calculated for nominally scaled variables (sex, pathogen, detection method, complication). For metric characteristics (age), median and mean with standard deviation as well as minimum and maximum [min;max] were determined. Data collection and statistical analysis were conducted with Microsoft Excel 2019 MSO (version 2303 build 16.0.16277.20202) RRID:SCR_016137.

## Results

### Patient collective

**Fig. 1:**
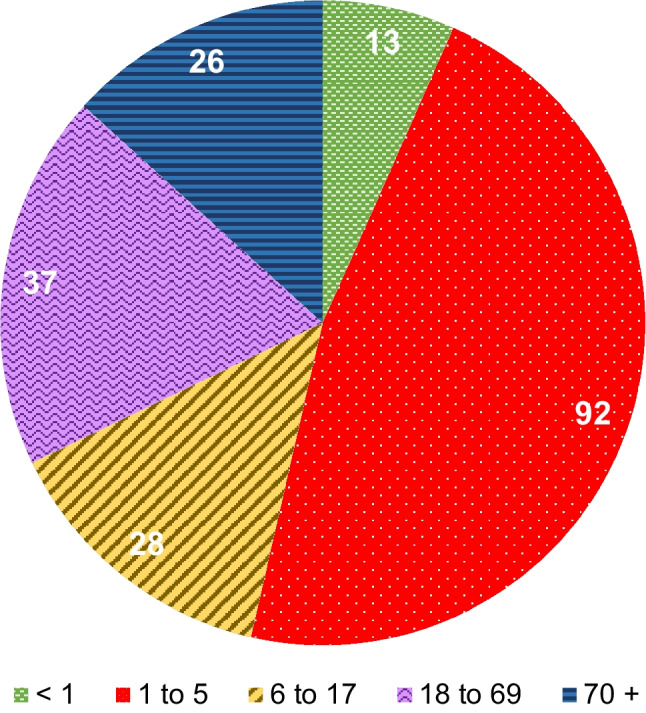
Distribution of age (2011 to 2023, *n* = 196). Digits in the pie chart represent the absolute numbers of the corresponding age group

In the period from 01/01/2011 to 30/06/2023 a total of 196 patients with unilateral AM were registered, including 133 children. 54% of the collective were children under the age of 6 years. After the age of 13 AM cases were regularly registered again only from the third decade of life onwards. Distribution of age and sex are shown in Fig. 1 and Table 1. In twelve patients (6%) preceding chronic otitis media was documented or incidentally found during radiodiagnostics or mastoidectomy. Four of them had perforation of the tympanic membrane and eight, including two children (11 and 13 years), had cholesteatoma.

**Table 1:** Characteristics of the collectives

	Total cases	Children	Adults
Number (%)	196 (100)	133 (68)	63 (32)
F/m (%)	81/115 (41/59)	53/80 (40/60)	28/35 (56/44)
Mean age [min;max]	21 ± 28.7 [0;93]	4 ± 3.0 [0;13]	63 ± 15.1 [21;93]
Median age	4	3	64

### Number of cases influenced by the COVID-19 pandemic

**Fig. 2:**
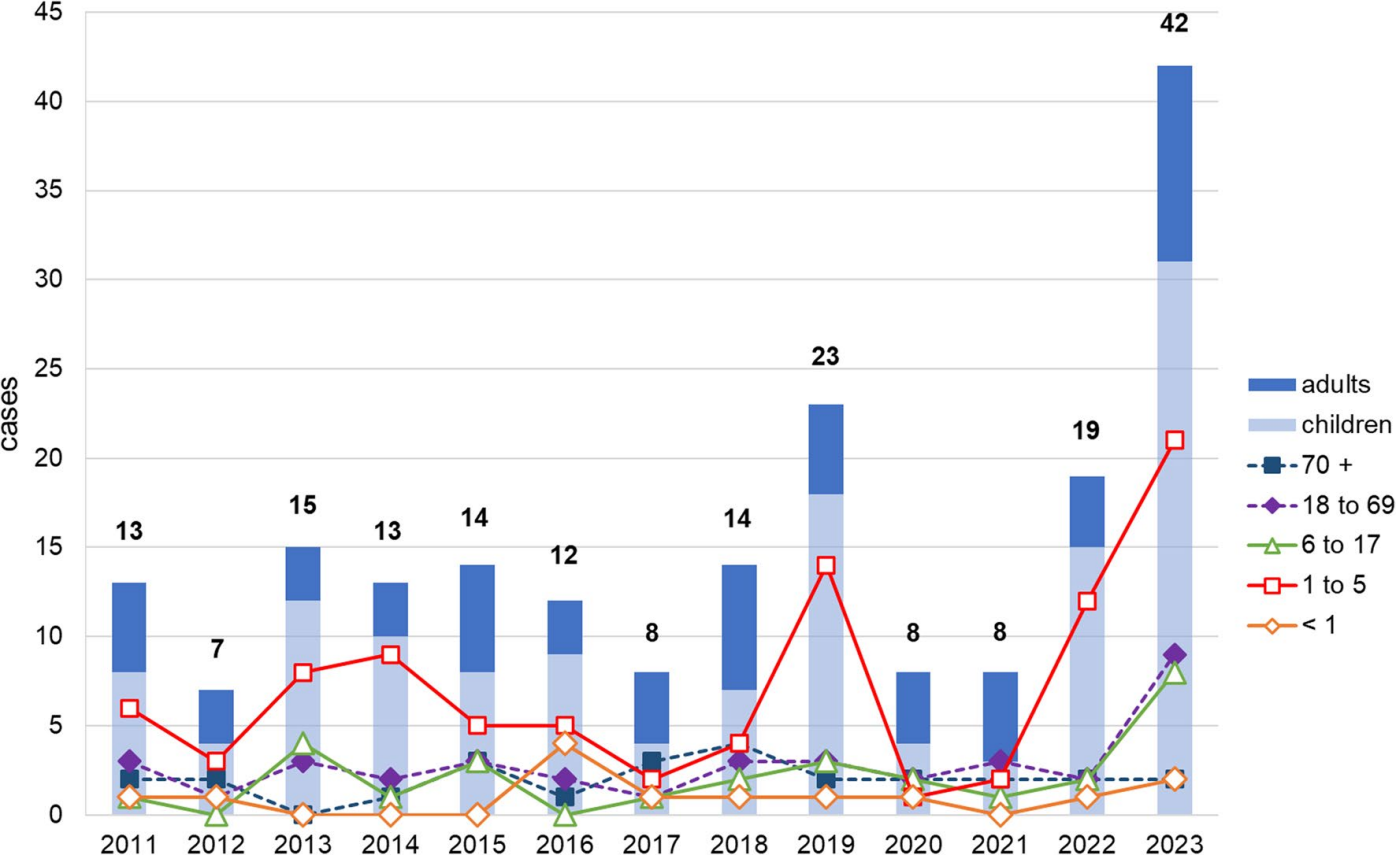
Distribution of cases differentiated by age over the study period. The digits above the bars describe the total number of cases per year. Cases in 2023 exclusively relate to the month of January to June

From 2011 to 2018 approximately one patient per month (7–15 per year) was treated for AM. In 2019 an unprecedented increase occurred with 23 cases per year. This increase was mainly caused by childhood infections with *S. pyogenes* (Fig. 2). During the COVID-19 pandemic from 2020 to 2021 the number of pediatric cases hospitalized reduced dramatically compared to adults, although similar total numbers were seen in 2012 and 2017. In 2022, after the reduction of NPI, a considerable surge of AM cases was observed.

Over the entire period, the number of cases of adult patients hardly changed. The first manifest increase of AM in adults was conspicuous from January 2023 on. Cases of newborns as well as children and adolescents aged 6 years and older did not show any relevant variation even under NPI. The major changes were detected in the age group of 1- to 5-year-old children (Fig. 2).

Patients with underlying chronic otitis media were treated for AM no more than one to two times per year throughout the entire study period. They did not affect the case numbers during and at the end of the pandemic.

### Seasonal course of AM infections

**Fig. 3:**
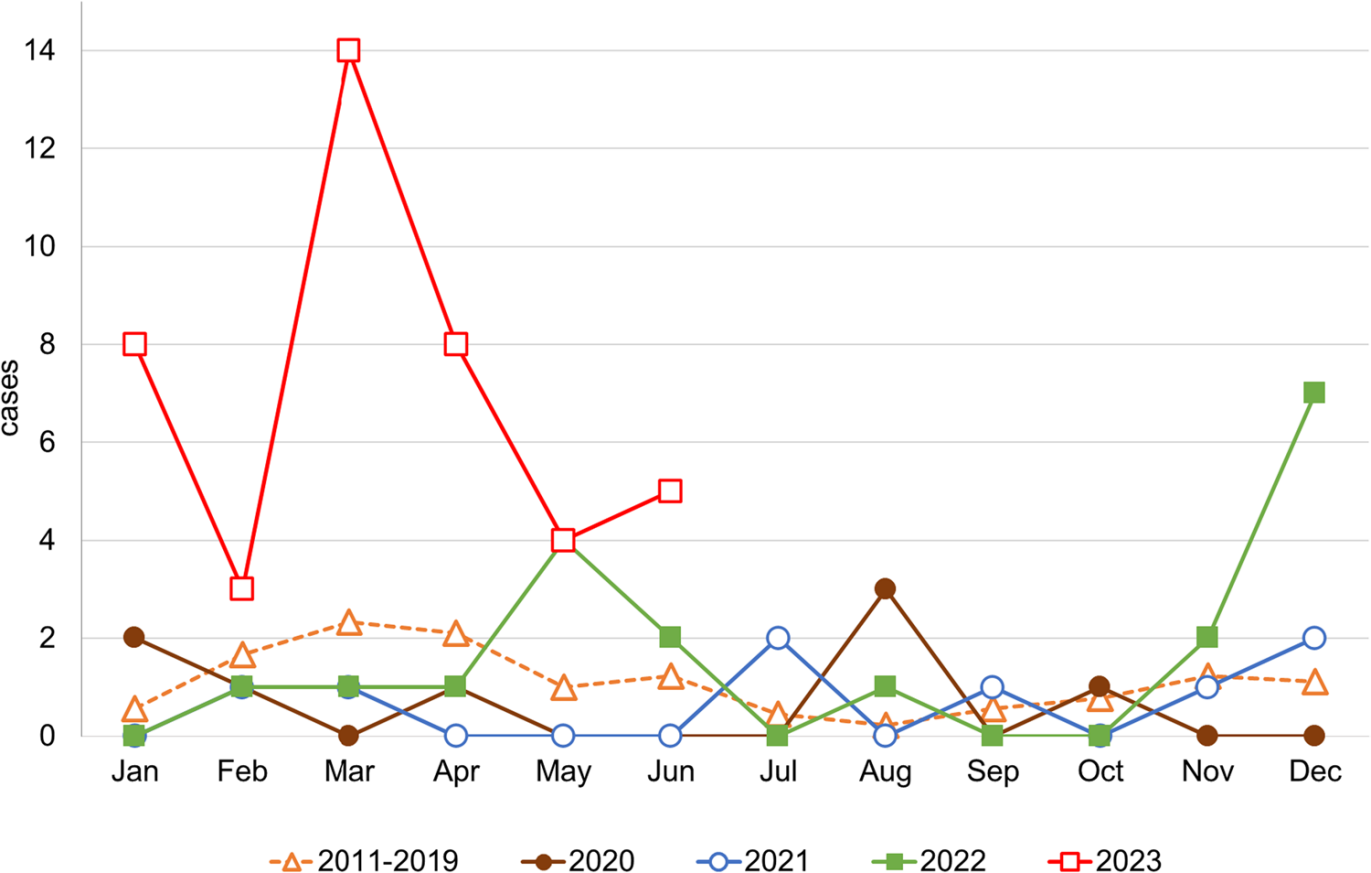
Seasonal development of infection cases. The broken line represents the mean values of the years 2011 to 2019 in the corresponding month. Values with continuous lines are absolute numbers

A seasonal accumulation of AM was evident from February to April and less pronounced from October to December until 2019. This variation was mainly caused by infections in children, whereas the number of cases in adult patients remained relatively stable over the year. The peak in spring was completely absent from 2020 to 2021 during the pandemic (Fig. 3). Adults and children were affected at similar rates. Only in December 2022 a steep rise in AM above pre-COVID levels was observed, which was primarily driven by childhood infections. Even infections in adults and adolescents increased more than ever before in the first half of 2023 (Figs. 2 and 3).

### Pathogens

Microbiological diagnostics were performed in 93% of all patients (*n* = 183). 169 swabs were taken during surgery. 39 microbiological probes originated from cerebrospinal fluid (CSF, *n* = 27) or blood cultures (BC, *n* = 12), in which *S. pneumoniae* was most frequently detected (*n*_CSF_ = 22, *n*_BC_ = 6). No bacteria were found in CSF cultures in one case and in BC in three cases. Pneumococci were the most common pathogens in both children and adults (*n* = 36 and 26 respectively; 29% and 43%). Infections in children were also frequently caused by *S. pyogenes* (*n* = 31; 25%; *n*_CSF_ = 3 and *n*_BC_ = 2), whereas this pathogen was detected only in seven adults (12%). *P. aeruginosa* caused twelve percent of AM in adults but only four percent in children. Pseudomonads occurred predominantly in cases with preexisting chronic otitis media or previous ear surgery. AM in chronic otitis media was also caused by Pneumococci, *S. aureus* and once by *S. pyogenes*. The distribution of pathogens per year is shown in Table 2.

**Table 2:** Annually detected pathogens in AM cases

Year of hospitalization	2011	2012	2013	2014	2015	2016	2017	2018	2019	2020	2021	2022	2023	Total (%)
Pathogen														
* S. pneumoniae*	***7***	***2***	2	***6***	***6***	***3***	***4***	***4***	5	***3***	***4***	***8***	8	**62** (34)
* S. pyogenes*	2	1	***3***	0	1	2	0	2	***9***	2	0	1	***15***	**38** (21)
* P. aeruginosa*	2	0	1	0	0	0	2	0	2	1	0	1	3	**12** (7)
* S. aureus*	0	0	1	0	1	2	1	1	1	1	0	0	0	**8** (4)
* H. influenzae*	0	1	1	1	0	0	0	0	0	0	0	0	2	**5** (3)
* F. necrophorum*	0	1	0	0	0	0	0	0	0	0	0	1	0	**2** (1)
Other	0	1	4	1	1	1	0	0	1	0	0	1	1	**11** (6)
Mixed flora	0	0	1	1	1	1	0	0	1	0	1	1	0	**7** (4)
No bacteria detected	1	1	2	4	3	3	1	4	3	0	3	4	5	**38** (21)
Total cases	**13**	**7**	**15**	**13**	**14**	**12**	**8**	**14**	**23**	**8**	**8**	**19**	**25**	

The most frequently detected pathogen is marked in bold italic. The cases in 2023 refer only to the months January to June

### Co-infections with SARS-CoV-2

From February 2020 to March 2023, all hospitalized patients were routinely screened for SARS-CoV-2 infection by PCR (*n* = 56). 37 patients received testing of more than three different viruses (panel testing). Infection with SARS-CoV-2 was detected in one case, influenza A and B in three cases, and metapneumovirus in one case.

### Complications

**Fig. 4:**
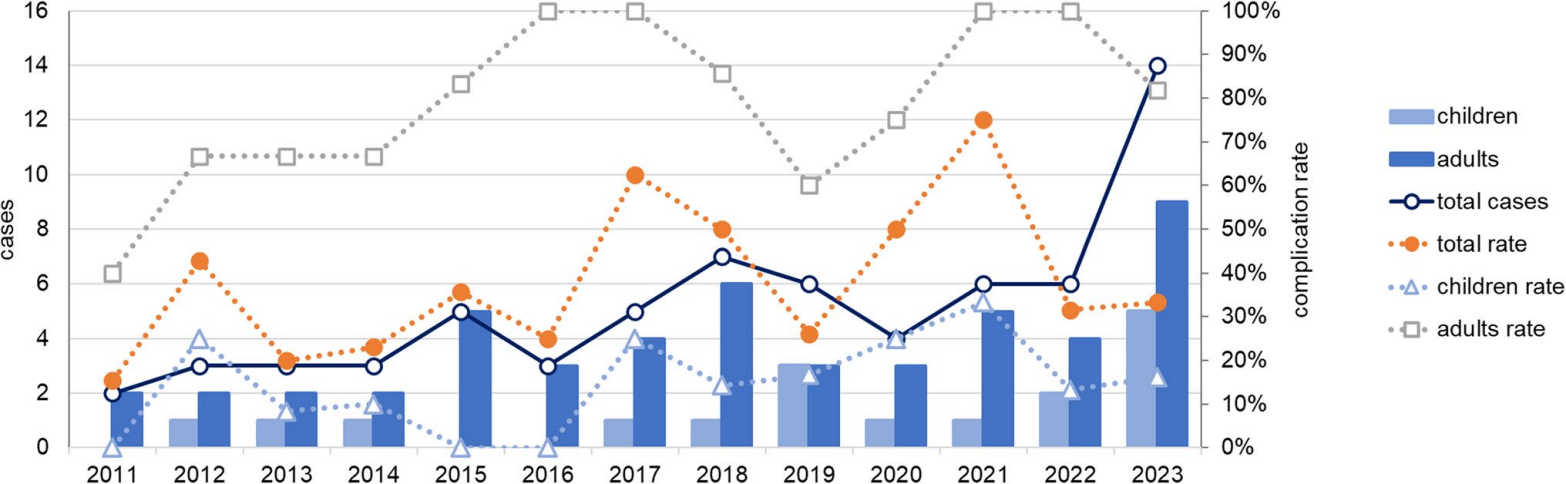
Acute mastoiditis with complication. Bars and blue continuous line represent absolute number of cases. Dotted lines depict the complication rates in relation to the respective population. Data from 2023 refer only to the month January to June

As a result of AM, 67 patients (34%) experienced an inflammatory complication as shown in Table 3. Ten patients suffered from two complications simultaneously, although meningitis was not considered as additional complication in the presence of a subdural empyema or brain abscess. The complication rate was 13% in children and 79% in adults. A trend toward a slow increase of complicated AM infections could be detected during the study period (Fig. 4). Throughout the COVID-19 pandemic, the complication rate was comparatively high in 2020 and 2021. The annual number of complicated courses in children varied even during the COVID-19 pandemic.

**Table 3:** Complications of acute mastoiditis

	Total cases (%)	Children (%)	Adults (%)
Complication			
Meningitis	40 (20)	6 (5)	34 (54)
Sinus vein thrombosis	12 (6)	9 (7)	3 (5)
Brain abscess	11 (6)	3 (2)	8 (13)
Facial nerve palsy	6 (3)	0 (0)	6 (10)
Bezold’s abscess	4 (2)	1 (1)	3 (5)
Other	4 (2)	3 (2)	1 (2)
Total	**77** (39)	**22** (17)	**55** (87)

*n* = 67; other: abducens nerve palsy, abscess of calvarium

Microbiological diagnostics were performed in 66 patients (99%). *S. pneumoniae* was most frequently detected, appearing in 28 of 77 complications (36%), and most often resulted in acute meningitis (23/28; 82%). The second most common germ causing complications was *S. pyogenes* (10/77; 13%). Four of six facial nerve palsies were associated with infections by *P. aeruginosa* (67%), with one occurring together with meningitis, but none with underlying cholesteatoma or chronic otitis media in the medical history. In the remaining documented complications different bacteria were found without predominance of any particular germ.

Mortality was 8% (5/63) in adults. Four patients died before the COVID-19 pandemic, one patient in 2021. Relevant underlying health conditions, such as chronic renal insufficiency, hepatic insufficiency, immunosuppression for rheumatoid arthritis, and diabetes mellitus, existed in all cases. The mean age at death was 71 ± 18.7 [52; 93]. Children were not affected.

## Discussion

### Age and sex

Infants and toddlers are at higher risk to develop an AM [2, 19–21] and anatomical as well as immunological considerations have been implicated as etiological factors [2, 6, 7, 21]. As also shown elsewhere [3, 4, 19, 22, 23], male patients were slightly more frequently affected than females. While children predominantly presented with the typical clinic of retroauricular redness and swelling, adults primarily demonstrated a complication of the AM, particularly meningitis [23]. These findings were similar in pre-, peri- and post-pandemic seasons (data not shown).

### Complications of AM

The complication rate of AM was 39% in total, with the caveat that in contrast to other studies SPA was not considered a complicated course, but a maximum clinical picture [2, 7, 20]. Here adults showed a much higher frequency compared to children. The rate in children (17%) was slightly higher than that reported in the literature [19]. Intracranial complications (meningitis, brain abscess, sinus vein thrombosis) were predominant at all ages. In the literature, rates varied between 4 and 16% in children [2, 19, 20, 22] and between 8 and 27.4% in adults [5, 23]. As in other reports, meningitis was the most common intracranial complication in adults [5, 23].

Over the study period, the incidences of complicated AM tended to increase (Fig. 4). The development of resistant bacterial strains due to inadequate antibiotic use, the shift of bacterial serotypes after the effective introduction of vaccines, or the low or late use of antibiotics for ear symptoms are possible causes [2, 24, 25]. The average complication rates during the COVID-19 pandemic were high, with 50% in 2020 and 75% in 2021. However, similarly high rates were observed in other years pre-pandemically (Fig. 4). Other studies described milder courses in children compared to pre-pandemic years, however considering only a short pre-pandemic period [12]. Despite the rapid increase in AM cases in 2023 the complication rate in both age groups was not substantially different from that of pre-pandemic years. During the pandemic SARS-CoV-2 co-infections were rarely detected in our collective. Consequently, these cases did not influence the course and severity of AM as had been previously speculated by other authors [12, 26]. Death was predominantly associated with relevant comorbidities in adults [5, 25]. Event of death occurred more frequently than in other studies [5, 23], which may be due to the much higher complication rate in our collective. Children were not affected by death, which was consistent to the literature [24].

### Spectrum of pathogens

As also suggested by other investigations, *S. pneumoniae* was the leading germ causing AM in children and adults in the present study [2, 4, 23, 24]. No pathogen was detected in 21% of the microbiological cultures, probably caused by antibiotic pre-treatment. *S. pyogenes* was recorded as the most common pathogen for three individual years. A periodic recurrence, as it is indicated in Table 2, is a common phenomenon [27]. However, it did not increase complication rates. In accordance with the literature, infections with *P. aeruginosa* occurred most frequently in patients with chronic ear infections and after a history of ear surgery [4, 5], while pseudomonades were particularly frequently associated with facial nerve palsy. In contrast to a British study [12], in which *P. aeruginosa* was predominantly detected peri-pandemically, *S. pneumoniae* was the dominant germ from 2020 until 2022 in the present collective. Interestingly, the steep increase of AM cases starting in December 2022 was induced by *S. pneumoniae* and *S. pyogenes*. These bacteria are responsible for various, seldom invasive infections that have been increasing since autumn 2022 in many European countries, including Germany, as well as the US [28–34]. The reasons for this increase are discussed below.

### Impact of the COVID-19 pandemic

Similar to other reports, pre-pandemically a seasonality of AM was evident in winter and spring, which approximately matched the seasonality of respiratory viruses, such as RSV and influenza [12, 35, 36].

In the course of the COVID-19 pandemic and the consecutive NPI the number of AM cases decreased from January 2020 until November 2022. This phenomenon was reported in several other countries, too [12, 16]. The decrease was followed by a kind of rebound from December 2022 on, for which there are several explanations.

As a result of the global spread of the SARS-CoV-2 virus, several preventive and protective public health measures were ordered at the beginning of 2020, considerably influencing the transmission of viral infections [37]. In Germany, NPI were agreed nationwide and differed only marginally in the first two years of the pandemic among the federal states. After repeated adjustments of the measures and restrictions the responsibility of implementing the measures was increasingly transferred to the federal states at the end of 2021. While phases of general lockdown and strict contact restrictions dominated in 2020, measures like wearing face masks, the obligation to vaccinate against the SARS-CoV-2 virus for certain occupational groups, and the regular testing before meetings and in public fora became increasingly important [38]. Thus, influenza and RSV were no longer detected in emergency departments either in children or in adults during the second wave of infections in the fall of 2020, and cases requiring inpatient treatment were held off [35, 36]. Similar observations were made in a worldwide prospective surveillance analysis regarding invasive infections with pneumococci, *Haemophilus influenzae* and meningococci [39]. Consistent with these findings, a relevant decrease in AM primarily caused by pneumococci and *S. pyogenes* occurred in the present analysis.

**Fig. 5:**
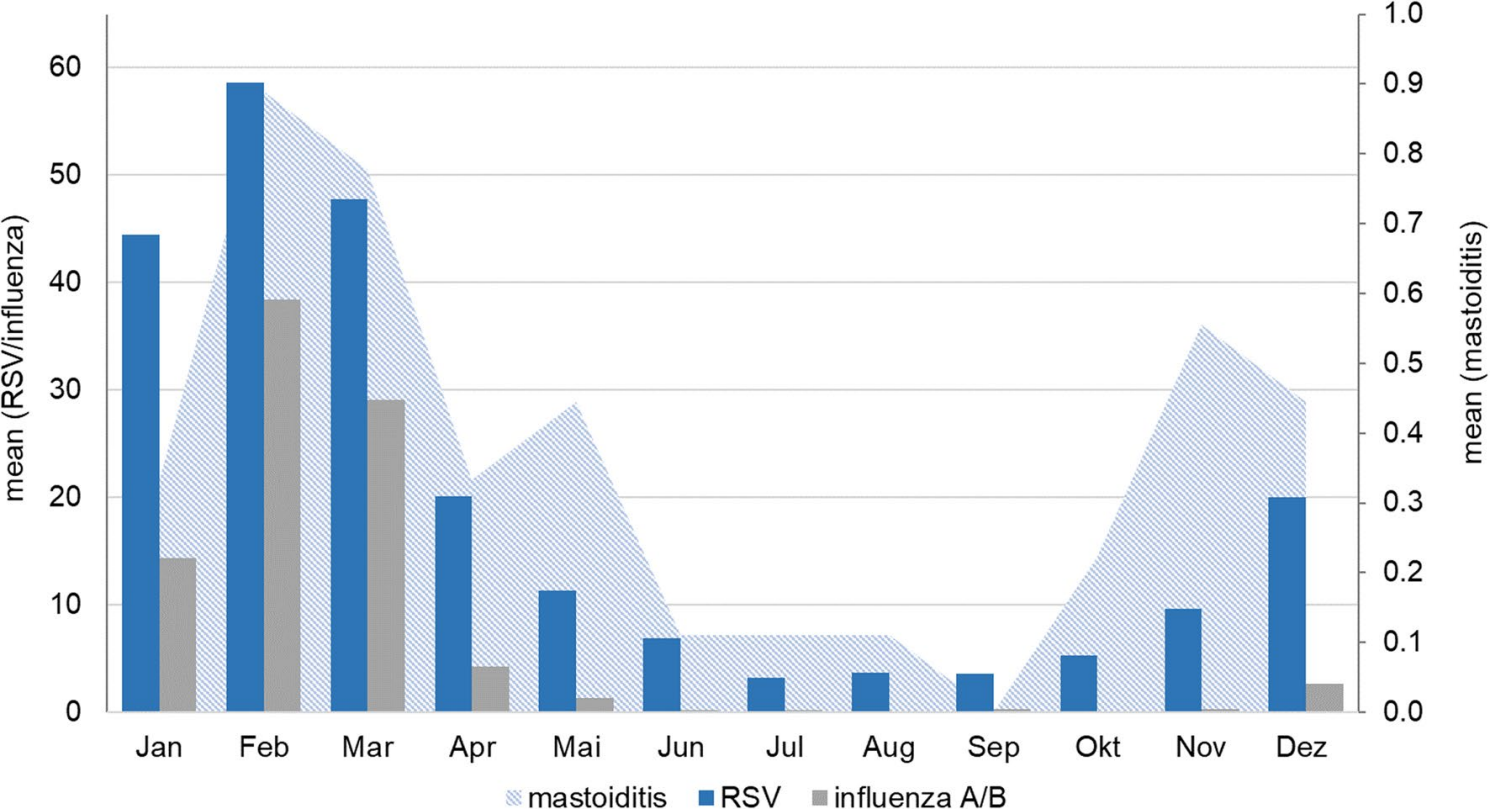
Periodicity of acute mastoiditis cases due to pneumococci (ENT department) and annual RSV and influenza detections (overall university hospital). Mean values of new cases per month in the period 2011 to 2019 are indicated

It is well known that infections with respiratory viruses pave the way for the outbreak of bacterial infections such as pneumococcal pneumonia by different mechanisms [40]. Selective examination of the pneumococcal cases of the present collective before the onset of the pandemic revealed seasonality similar to that observed for RSV and influenza infections (Fig. 5).

Recently, the complete absence of the RSV season 2020/21 in three large pediatric hospitals in northern Germany was reported, and the consecutive risk for increasing severe infections in the following season due to the current omission of risky primary infections was discussed [35]. Subsequently, a remarkable increase of relevant respiratory infections was recorded starting in June 2021 [41]. The situation was different for the registered influenza cases in Germany, which increased only slightly in the flu season 2021/22 but rose substantially earlier and higher than before the pandemic in the season 2022/23 [42]. At this time, starting from October 2022, all mandatory measures in Germany were eliminated and given to the federal states as tools adapted to their needs. Especially children and adolescents were affected more frequently by influenza compared to previous years [42]. In line with these changes in the present study a massive increase of AM has been observed since the end of 2022 affecting especially young children and adults. However, the direct detection of viral co-infections was only successful in individual cases. Since the accumulation of AM cases was detected in 2022 for the first time and not in 2021, as it was the case for RSV, it must be assumed that other factors than the rise in respiratory virus infections may play a role, too. The lack of exposure of the immune system to pathogens due to the NPI and the consecutive progress in severe infections after lifting or reduction of NPI have been discussed especially in children [43, 44]. The global decline of standard immunizations in children during the COVID-19 pandemic, particularly during the lockdown phases, may have played an additional role [44, 45]. The concept of an “immune dept” or “immunity gap” is controversial discussed, especially in the lay press [46]. However, the fact that the incidence of diseases transmitted by droplets or direct contact excessively increased after the relaxation and reversal of many NPI [43] suggests an association and could be also responsible, at least in parts, for the increase in AM in children detected in the present study.

Even more frequently than pneumococci, group A streptococci (GAS, *S. pyogenes*) were detected in microbiological probes of AM cases in 2023. In line, several European Countries reported an ongoing increase of invasive GAS infections particularly in children starting in autumn 2022 [28, 29, 32–34, 47–49]. Similarly, the CDC advised against the increasing number of invasive GAS infections in fall 2022 in many regions across the US, which was higher than before the pandemic [30]. So far an association with the development of new or resistant bacterial strains could not be found [32, 33, 47, 50]. With regards to Germany first evaluations indicate that, in line with the present AM data, substantially fewer GAS infections were registered during the pandemic, and that cases of invasive infections have escalated in all age groups since the fourth quarter of 2022 [31]. According to the Robert-Koch-Institute (RKI) this also applies to invasive infections caused by pneumococci and *Haemophilus influenzae* [31]*.* However, RKI’s data demonstrated that the peak in invasive bacterial infections in the fourth quarter of 2022 affected all age groups but particularly frequently adults more than 65 years old. This is in contrast to the observations in the present monocentric study and to other notifications in Europe, in which younger children were predominantly affected [32, 33].

Finally, drug shortages for antibiotic suspensions, as they have existed since late 2022, may also be a secondary cofactor for the observed increase of invasive and complicated infections [51].

## Limitations

The retrospective, monocentric study design has some limitations. Cases from surrounding pediatric hospitals, which tend to treat AM conservatively and refer patients only if they do not respond to treatment, were not included, so complication rates may have been significantly higher in the present population. Second, the inclusion criteria may have introduced some bias, as cases diagnosed with prolonged or complicated AOM and not encoded as “acute mastoiditis” or “mastoiditis unspecified”, which are likely to have similar clinical signs as defined for AM, were not included in the study. Furthermore, patients without typical clinical signs of acute mastoiditis—such as redness and retroauricular swelling—were diagnosed by radiodiagnostics alone, but were also classified as having typical AM. Intracranial complications may therefore be over-represented in the collective. As the analysis did not differentiate between bacterial strains, the development of resistant strains after the pandemic cannot be excluded as a causal factor for the described "rebound".

## Conclusion

Most of the NPI have been shown to be effective against the transmission of respiratory infections, secondarily lowering AM cases in the described collective. The rapid increase in viral respiratory disorders after the reduction of major pandemic restrictions and the reduced peri-pandemic exposure of the immune system to infectious agents particularly in children appear to trigger invasive bacterial infections and explain the steep rise in AM since December 2022. Taking this into account the need for regular vaccination of children and adults must be emphasized also and especially during the slowly ending pandemic. Furthermore, even if generally ordered pandemic-like restrictions are not recommended, selected measures like wearing face masks can effectively prevent the spread of infections in case of a present cold and thus also influence the development of AOM and—depending on the virulence of the pathogen and the predisposing factors—AM. The impact of universally mandated NPI on the seasonal rhythmicity of certain respiratory pathogens should be considered in the future, also because it could lead to their shifting and intensification. Informing and educating people about the spread of communicable respiratory diseases and the development of invasive bacterial infections should be brought into greater focus.

## Data Availability

The data that support the findings of this study are available on request from the corresponding author.

## References

[1] Mierzwiński J, Tyra J, Haber K et al (2019) Therapeutic approach to pediatric acute mastoiditis—an update. Braz J Otorhinolaryngol 85:724–732. 10.1016/j.bjorl.2018.06.002 30056031 10.1016/j.bjorl.2018.06.002 PMC9443014

[2] Cassano P, Ciprandi G, Passali D (2020) Acute mastoiditis in children. Acta Biomed 91:54–59. 10.2375/abm.v91i1-S.9259 32073562 10.2375/abm.v91i1-S.9259 PMC7947742

[3] Garcia C, Salgueiro AB, Luís C et al (2017) Acute mastoiditis in children: Middle ear cultures may help in reducing use of broad spectrum antibiotics. Int J Pediatr Otorhinolaryngol 92:32–37. 10.1016/j.ijporl.2016.11.002 28012530 10.1016/j.ijporl.2016.11.002

[4] Laulajainen-Hongisto A, Saat R, Lempinen L et al (2014) Bacteriology in relation to clinical findings and treatment of acute mastoiditis in children. Int J Pediatr Otorhinolaryngol 78:2072–2078. 10.1016/j.ijporl.2014.09.007 25281339 10.1016/j.ijporl.2014.09.007

[5] Laulajainen-Hongisto A, Jero J, Markkola A et al (2016) Severe acute otitis media and acute mastoiditis in adults. J Int Adv Otol 12:224–230. 10.5152/iao.2016.2620 27895000 10.5152/iao.2016.2620

[6] Obringer E, Chen JL (2016) Acute mastoiditis caused by streptococcus pneumoniae. Pediatr Ann 45:e176–e179. 10.3928/00904481-20160328-01 27171806 10.3928/00904481-20160328-01

[7] Loh R, Phua M, Shaw CKL (2018) Management of paediatric acute mastoiditis: Systematic review. J Laryngol Otol 132:96–104. 10.1017/S0022215117001840 28879826 10.1017/S0022215117001840

[8] Anne S, Schwartz S, Ishman SL et al (2019) Medical versus surgical treatment of pediatric acute mastoiditis: a systematic review. Laryngoscope 129:754–760. 10.1002/lary.27462 30284265 10.1002/lary.27462

[9] Xiao J, Dai J, Hu J et al (2021) Co-benefits of nonpharmaceutical intervention against COVID-19 on infectious diseases in China: a large population-based observational study. Lancet Reg Heal - West Pacific. 10.1016/j.lanwpc.2021.100282 10.1016/j.lanwpc.2021.100282 PMC8484818 34611630

[10] Ullrich A, Schranz M, Rexroth U et al (2021) Impact of the COVID-19 pandemic and associated non-pharmaceutical interventions on other notifiable infectious diseases in Germany: an analysis of national surveillance data during week 1–2016 – week 32–2020. Lancet Reg Heal - Eur. 10.1016/j.lanepe.2021.100103 10.1016/j.lanepe.2021.100103 PMC8454829 34557831

[11] Völk S, Pfirrmann M, Koedel U et al (2022) Decline in the number of patients with meningitis in German hospitals during the COVID-19 pandemic. J Neurol 269:3389–3399. 10.1007/s00415-022-11034-w 35316388 10.1007/s00415-022-11034-w PMC8938731

[12] Smith ME, Jones GH, Hardman JC et al (2022) Acute paediatric mastoiditis in the UK before and during the COVID-19 pandemic: a national observational study. Clin Otolaryngol 47:120–130. 10.1111/coa.13869 34606691 10.1111/coa.13869 PMC8652842

[13] Marom T, Pitaro J, Shah UK et al (2022) Otitis Media Practice During the COVID-19 Pandemic. Front Cell Infect Microbiol. 10.3389/fcimb.2021.749911 35071032 10.3389/fcimb.2021.749911 PMC8777025

[14] Torres-García L, Acosta RM, Martínez AC et al (2022) Evolution in the incidence of infectious diseases in the pediatric ENT area during the COVID-19 pandemic. Acta Otorrinolaringol (English Ed). 10.1016/j.otoeng.2022.11.007 10.1016/j.otoeng.2022.11.007 PMC9678834 36427791

[15] Quraishi N, Ray M, Srivastava R et al (2022) A multicentre retrospective cohort study on COVID-19-related physical interventions and adult hospital admissions for ENT infections. Eur Arch Oto-Rhino-Laryngol 279:2671–2678. 10.1007/s00405-021-07180-y 10.1007/s00405-021-07180-y PMC8607061 34807284

[16] Jesus CR, Rosa AAS, Meneses AS et al (2021) Impact of social distancing in response to COVID-19 on hospitalizations for laryngitis, tracheitis, otitis media, and mastoiditis in children aged 0 to 9 years in Brazil. J Bras Pneumol 47:e20210229. 10.36416/1806-3756/e20210229 34909923 10.36416/1806-3756/e20210229 PMC8836655

[17] Pruccoli G, Castagno E, Raffaldi I et al (2023) The importance of RSV epidemiological surveillance: a multicenter observational study of RSV infection during the COVID-19 pandemic. Viruses 15:280. 10.3390/v15020280 36851494 10.3390/v15020280 PMC9963567

[18] Curatola A, Graglia B, Ferretti S et al (2023) The acute bronchiolitis rebound in children after COVID-19 restrictions: a retrospective, observational analysis. Acta Biomed. 10.23750/abm.v94i1.13552 36786263 10.23750/abm.v94i1.13552 PMC9987502

[19] Spratley J, Silveira H, Alvarez I, Pais-Clemente M (2000) Acute mastoiditis in children: review of the current status. Int J Pediatr Otorhinolaryngol 56:33–40 11074113 10.1016/S0165-5876(00)00406-7

[20] Favre N, Patel VA, Carr MM (2021) Complications in pediatric acute mastoiditis: HCUP KID analysis. Otolaryngol 165:722–730. 10.1177/0194599821989633 10.1177/0194599821989633 33588620

[21] Sokolov M, Tzelnick S, Stern S et al (2021) Acute mastoiditis in infants younger than 6 months: is an alternative treatment protocol needed? Eur Arch Oto-Rhino-Laryngol 278:339–344. 10.1007/s00405-020-06088-3 10.1007/s00405-020-06088-3 32500325

[22] Grossman Z, Zehavi Y, Leibovitz E et al (2016) Severe acute mastoiditis admission is not related to delayed antibiotic treatment for antecedent acute otitis media. Pediatr Infect Dis J 35:162–165. 10.1097/INF.0000000000000951 26461229 10.1097/INF.0000000000000951

[23] Palma S, Bovo R, Benatti A et al (2014) Mastoiditis in adults: a 19-year retrospective study. Eur Arch Oto-Rhino-Laryngol 271:925–931. 10.1007/s00405-013-2454-8 10.1007/s00405-013-2454-8 23589156

[24] Ibrahim SI, Cheang PP, Nunez DA (2010) Incidence of meningitis secondary to suppurative otitis media in adults. J Laryngol Otol 124:1158–1161. 10.1017/S0022215110000976 20441675 10.1017/S0022215110000976

[25] Koelman DLH, Brouwer MC, van de Beek D (2020) Resurgence of pneumococcal meningitis in Europe and Northern America. Clin Microbiol Infect 26:199–204. 10.1016/j.cmi.2019.04.032 31100424 10.1016/j.cmi.2019.04.032

[26] Enrique GL, Margarita BB, Ángel MJ et al (2021) COVID-19 and severe ENT infections in pediatric patients. IS there a relationship? Int J Pediatr Otorhinolaryngol. 10.1016/j.ijporl.2021.110714 33894522 10.1016/j.ijporl.2021.110714 PMC8051009

[27] Laakso JT, Rissanen V, Ruotsalainen E et al (2021) Severe acute otitis media and mastoiditis caused by group A beta-hemolytic streptococcus. Laryngoscope Investig Otolaryngol 6:1158–1166. 10.1002/lio2.659 34667861 10.1002/lio2.659 PMC8513450

[28] World Health Organization (WHO) (2022) Disease Outbreak News: Increased incidence of scarlet fever and invasive Group A Streptococcus infection—multi-country. https://www.who.int/emergencies/disease-outbreak-news/item/2022-DON429. Accessed 15 Jun 2023

[29] European Centre for Disease Prevention and Control (ECDC) (2022) Weekly Communicable Disease Threats Report, Week 49, 4–10 December. https://www.ecdc.europa.eu/sites/default/files/documents/ECDC Weekly Communicable Disease Threats Report 2022w49.pdf. Accessed 15 Jun 2023

[30] CDC (2023) Increase in Invasive Group A Strep Infections, 2022–2023. https://www.cdc.gov/groupastrep/igas-infections-investigation.html#print. Accessed 15 Jun 2023

[31] Robert Koch Institut (2023) Update: anstieg bakterieller Infektionen durch Gruppe-A-Streptokokken, Pneumokokken und Haemophilus influenzae in Deutschland seit Ende 2022. In: Epidemiol. Bull. https://www.rki.de/DE/Content/Infekt/EpidBull/Archiv/2023/Ausgaben/08_23.pdf?__blob=publicationFile. Accessed 19 Aug 2023

[32] Guy R, Henderson KL, Coelho J et al (2023) Increase in invasive group A streptococcal infection notifications, England, 2022. Euro Surveill. 10.2807/1560-7917.ES.2023.28.1.2200942 36695450 10.2807/1560-7917.ES.2023.28.1.2200942 PMC9817207

[33] Johannesen TB, Munkstrup C, Edslev SM et al (2023) Increase in invasive group A streptococcal infections and emergence of novel, rapidly expanding sub-lineage of the virulent Streptococcus pyogenes M1 clone, Denmark, 2023. Eurosurveillance. 10.2807/1560-7917.ES.2023.28.26.2300291 37382884 10.2807/1560-7917.ES.2023.28.26.2300291 PMC10311951

[34] Lassoued Y, Assad Z, Ouldali N et al (2023) Unexpected increase in invasive group a streptococcal infections in children after respiratory viruses outbreak in France: a 15-year time-series analysis. Open Forum Infect Dis. 10.1093/ofid/ofad188 37180594 10.1093/ofid/ofad188 PMC10167988

[35] Lange M, Happle C, Hamel J et al (2021) Non-appearance of the RSV season 2020/21 during the COVID-19 pandemic-prospective, multicenter data on the incidence of respiratory syncytial virus (RSV) infection. Dtsch Arztebl Int 118:561–562. 10.3238/arztebl.m2021.0300 34725031 10.3238/arztebl.m2021.0300 PMC8579425

[36] Stamm P, Sagoschen I, Weise K et al (2021) Influenza and RSV incidence during COVID-19 pandemic—an observational study from in-hospital point-of-care testing. Med Microbiol Immunol 210:277–282. 10.1007/s00430-021-00720-7 34604931 10.1007/s00430-021-00720-7 PMC8487758

[37] Shen Y, Powell G, Ganser I et al (2021) Monitoring non-pharmaceutical public health interventions during the COVID-19 pandemic. Sci Data. 10.1038/s41597-021-01001-x 34429423 10.1038/s41597-021-01001-x PMC8385050

[38] Grote U, Arvand M, Brinkwirth S et al (2021) Measures to cope with the COVID-19 pandemic in Germany: nonpharmaceutical and pharmaceutical interventions. Bundesgesundheitsblatt - Gesundheitsforsch - Gesundheitsschutz 64:435–445 10.1007/s00103-021-03306-z PMC8010780 33787944

[39] Brueggemann AB, Jansen van Rensburg MJ, Shaw D et al (2021) Changes in the incidence of invasive disease due to *Streptococcus**pneumoniae*, *Haemophilus**influenzae*, and *Neisseria**meningitidis* during the COVID-19 pandemic in 26 countries and territories in the Invasive Respiratory Infection Surveillance Initiative: a prospective analysis of surveillance data. Lancet Digit Heal 3:e360–e370. 10.1016/S2589-7500(21)00077-7 10.1016/S2589-7500(21)00077-7 PMC8166576 34045002

[40] Moore DP, Dagan R, Madhi SA (2012) Respiratory viral and pneumococcal coinfection of the respiratory tract: implications of pneumococcal vaccination. Expert Rev Respir Med 6:451–465. 10.1586/ers.12.32 22971069 10.1586/ers.12.32

[41] Maison N, Peck A, Illi S et al (2022) The rising of old foes: impact of lockdown periods on “non-SARS-CoV-2” viral respiratory and gastrointestinal infections. Infection 50:519–524. 10.1007/s15010-022-01756-4 35076891 10.1007/s15010-022-01756-4 PMC8787179

[42] Nationale Lenkungsgruppe Impfen (2023) Monitoring & daten: influenza. In: https://www.nali-impfen.de/monitoring-daten/krankheitsfaelle-in-deutschland/influenza/. https://www.nali-impfen.de/monitoring-daten/krankheitsfaelle-in-deutschland/influenza/. Accessed 15 Jun 2023

[43] Cohen PR, Rybak A, Werner A et al (2022) Trends in pediatric ambulatory community acquired infections before and during COVID-19 pandemic: a prospective multicentric surveillance study in France. Lancet Reg Heal Eur 22:100497. 10.1016/j.lanepe.2022.100497 10.1016/j.lanepe.2022.100497 PMC9398201 36034052

[44] Oh K-B, Doherty TM, Vetter V, Bonanni P (2022) Lifting non-pharmaceutical interventions following the COVID-19 pandemic—the quiet before the storm? Expert Rev Vaccines 21:1541–1553. 10.1080/14760584.2022.2117693 36039786 10.1080/14760584.2022.2117693

[45] Tan L LJ, Safadi MAP, Horn M, et al (2023) Pandemic’s influence on parents’ attitudes and behaviors toward meningococcal vaccination. Hum Vaccin Immunother. 10.1080/21645515.2023.2179840 10.1080/21645515.2023.2179840 PMC10026861 36883777

[46] Cohen R, Levy C, Rybak A et al (2023) Immune debt: Recrudescence of disease and confirmation of a contested concept. Infect Dis Now 53:104638. 10.1016/j.idnow.2022.12.003 36535583 10.1016/j.idnow.2022.12.003 PMC9756601

[47] de Gier B, Marchal N, de Beer-Schuurman I et al (2023) Increase in invasive group A streptococcal (*Streptococcus**pyogenes*) infections (iGAS) in young children in the Netherlands, 2022. Eurosurveillance. 10.2807/1560-7917.ES.2023.28.1.2200941 36695447 10.2807/1560-7917.ES.2023.28.1.2200941 PMC9817208

[48] UK Health Security Agency (UKHSA) (2022) Group A streptococcal infections: first update on seasonal activity in England, 2022 to 2023 - GOV.UK. In: UKHSA. https://www.gov.uk/government/publications/group-a-streptococcal-infections-activity-during-the-2022-to-2023-season/group-a-streptococcal-infections-report-on-seasonal-activity-in-england-2022-to-2023. Accessed 15 Jun 2023

[49] van Kempen EB, Bruijning-Verhagen PCJ, Borensztajn D et al (2023) Increase in invasive group a streptococcal infections in children in the Netherlands, a survey among 7 hospitals in 2022. Pediatr Infect Dis J 42:e122–e124. 10.1097/INF.0000000000003810 36728741 10.1097/INF.0000000000003810

[50] European Centre for Disease Prevention and Control (ECDC) (2023) Weekly communicable disease threats report, week 14, 2–8 April 2023. https://www.ecdc.europa.eu/sites/default/files/documents/Communicable-Disease-Threats-Report-2-8-April-2023-Week-14.pdf. Accessed 18 Jun 2023

[51] U.S. Food & Drug Administration (2023) FDA drug shortage: amoxicillin. In: https://www.accessdata.fda.gov/scripts/drugshortages/dsp_ActiveIngredientDetails.cfm?AI=Amoxicillin%20Oral%20Powder%20for%20Suspension&st=c&tab=tabs-1

